# Socioeconomic and psychosocial predictors of dental healthcare use among Brazilian preschool children

**DOI:** 10.1186/1472-6831-13-60

**Published:** 2013-10-31

**Authors:** Rômulo Vaz Machry, Simone Tuchtenhagen, Bernardo Antonio Agostini, Carlos Roberto da Silva Teixeira, Chaiana Piovesan, Fausto Medeiros Mendes, Thiago Machado Ardenghi

**Affiliations:** 1 Department of Stomatology, Federal University of Santa Maria, UFSM, Rua Cel. Niederauer 917/208, Santa Maria, RS, Brazil; 2 Centro Universitário Franciscano (UNIFRA), Santa Maria, RS, Brazil; 3 Department of Pediatric Dentistry, University of São Paulo, USP-SP, São Paulo, Brazil

**Keywords:** Dental care, Healthcare disparities, Oral health, Preschool children, Health perceptions

## Abstract

**Background:**

Disparities in utilization of oral healthcare services have been attributed to socioeconomic and individual behavioral factors. Parents’ socioeconomic status, demographics, schooling, and perceptions of oral health may influence their children’s use of dental services. This cross-sectional study assessed the relationships between socioeconomic and psychosocial factors and the utilization of dental health services by children aged 1–5 years.

**Methods:**

Data were collected through clinical exams and a structured questionnaire administered during the National Day of Children’s Vaccination. A Poisson regression model was used to estimate prevalence ratios and 95% confidence intervals.

**Results:**

Data were collected from a total of 478 children. Only 112 (23.68%) were found to have visited a dentist; 67.77% of those had seen the dentist for preventive care. Most (63.11%) used public rather than private services. The use of dental services varied according to parental socioeconomic status; children from low socioeconomic backgrounds and those whose parents rated their oral health as “poor” used dental services less frequently. The reason for visiting the dentist also varied with socioeconomic status, in that children of parents with poor socioeconomic status and who reported their child’s oral health as “fair/poor” were less likely to have visited the dentist for preventive care.

**Conclusion:**

This study demonstrated that psychosocial and socioeconomic factors are important predictors of the utilization of dental care services.

## Background

Disparities in oral healthcare utilization have been attributed to socioeconomic and individual behavioral factors [1-5]. However, in most developing countries, data are scarce regarding children’s use of dental care services [2].

In Brazil, the latest national population-based oral health study showed that 18.1% of children age 12 years had never visited a dentist [6]. The same study reported regional inequalities in the use of dental services indicating that the most economically developed regions had the highest proportion of children who had received dental care in the previous year. It is strongly recommended that children see a dentist as early as 6 months of age, and no later than 6 months after the first tooth erupts [7,8]. However, 77.9% of children in Brazil have never visited a dentist. Thus, identifying the determinants of the utilization of dental healthcare services is essential for the development and improvement of public health policies in this country [9,10].

There is considerable evidence that the use of dental care services may be influenced by socioeconomic and psychosocial factors [4,10]. Previous studies have reported that parents’ perceptions of their child’s oral health could influence oral health decisions and patterns of healthcare for children [10,11]. However, little data is available regarding the interaction of different predictors of dental care utilization in Brazilian preschool children [2].

This cross-sectional study assessed the relationships between socioeconomic and psychosocial factors and the utilization of dental health services by children aged 1–5 years.

## Methods

### Ethics

This study was approved by the Ethics in Research Committee of the Federal University of Santa Maria in Santa Maria, Brazil. A letter was given to all parents explaining the aims of the study and asking for their consent for their children’s participation. Consent was obtained from all parents before data collection.

### Sample

A questionnaire-based survey was administered to parents of 1–5-year-old children from Santa Maria, Rio Grande do Sul, Brazil. Santa Maria is a medium-sized city located in the south of Brazil with an estimated population of 261,031 inhabitants, including 18,420 children aged 0–5 years [12]. The following parameters were adopted to determine a sample size appropriate for assessing the association between the use of dental care services and various independent variables: 5% standard error, 80% power, 95% confidence interval, 10% non-response rate, 2:1 ratio of unexposed to exposed, and a prevalence ratio to be detected of at least 1.8. As we used multistage rather than simple random sampling, respondents tended to be clustered; thus, an adjustment for the sample design of 1.4 was adopted (design effect). The minimum sample size was estimated at 456 children.

### Data collection

The study was conducted with children who attended the National Day of Children’s Vaccination. More than 97% of children living in the city participated in the vaccination program. A sampling quota was selected from among all children who visited health centers in the municipality of Santa Maria. Health centers were used as sampling points because the city is divided into 5 administrative regions, and each has public health centers that are responsible for vaccinating the children who live in that area. For this study, all health centers that possessed a dental chair (15 health centers) were used as sample points. These were the largest health centers in the city; almost 90% of children visited these centers when this study was conducted. The sample was stratified according to the number of children in each area. During the survey, every fifth child in the queue for vaccination was invited to participate in the study. If their guardians did not consent to participation, the next child in the queue was selected. The same selection procedure was followed at all 15 participating health centers.

Data were collected through clinical examinations and a structured questionnaire administered by 15 researchers and 30 assistants who had been trained and calibrated prior to data collection. The training included theoretical explanations and informative discussions facilitated by clinical photographic examples. Subsequently, all examiners performed an examination of 60 exfoliated primary teeth set in arch models, aided by a dental operating light, 3-in-1 syringe, plane dental mirror, and a WHO periodontal probe. After the in vitro sessions, 10 children were examined twice by all examiners, at an interval of 1 week between examinations. Intra- and inter-examiner reliabilities were assessed; a total of 36 hours was spent on training and calibration. A benchmark dental examiner conducted the entire training and calibration process. Values for inter- and intra-observer agreement for ICDAS scores ranged from 0.86 to 0.92 and from 0.77 to 0.94, respectively.

Children were examined while seated on a dental chair under conventional dental illumination. Visual examinations for ICDAS criteria were conducted with plane dental mirrors and WHO periodontal probes. Wet gauze pads, periodontal probes, toothbrushes, and dental floss were used to remove surface dental plaque [13]. As the ICDAS has demonstrated comparability with standard criteria (WHO) in an epidemiological survey of preschool children [14], we used the ICDAS cut-off point of 3 (0–2 sound, 3–6 carious) to calculate the number of decayed/missing/filled teeth (dmft). The prevalence of dental caries was considered as children with dmft ≥ 1.

A structured questionnaire was used to collect data for variables including age, children’s gender and race, family income, parents’ educational level, and health behaviors. Socioeconomic status was measured in terms of household income and parents’ educational level. Household income was measured in terms of the Brazilian Minimum Wage (BMW), a common standard for this type of assessment, which corresponded to approximately $300 US during the data collection period. The threshold used was based in the distribution of our data. Therefore, we used 1 BMW as an income threshold because this value corresponded to the median value of our data. Educational level compared those fathers and mothers who had completed eight years of formal instruction, which corresponds to primary school in Brazil, with those who had completed only lower education (less than eight years of formal education). Parents answered questions about children’s tooth brushing frequency; children who brushed their teeth 3 or more times per day were compared with those who brushed less often. Data on parents’ perceptions of their child’s oral health were measured by the following questions: (1) “*Would you say that your child’s oral health is 1 (excellent), 2 (good), 3 (fair), or 4 (poor)*?” For analysis, responses were dichotomized into good (scores 1 and 2) and poor (scores 3 and 4) oral health. The feasibility of the questionnaire was previously assessed in a sample of 20 parents during the calibration process.

Our primary outcome was the use of dental services as measured by the question “Has your child ever visited the dentist?” When applicable, we inquired about the reason for the visit (preventive or non-preventive) and the type of service utilized (public or private).

### Statistical analyses

Data were analyzed using STATA 12.0 (Stata Corp., College Station, TX, USA). Two outcomes were analyzed: prevalence of children who had never visited a dentist and the reason for the visit (preventive/non-preventive). Multivariate Poisson regression considering the cluster design was performed to assess the association between the predictor variables and the outcomes. A backward stepwise procedure was used to include or exclude explanatory variables in the models. Explanatory variables that correlated with each outcome with a P value ≤0.20 (unadjusted analyses) were included in the multivariate analysis. Only explanatory variables with a P value ≤0.05 after adjustment were selected for the final models.

## Results

A total of 478 children—232 boys (48.54%) and 246 girls (52.46%)—participated in this study. Most were 36–59 months old (61.09%) and of white skin color (79.29%). Indicators of parental education and occupation were similar: nearly 56% of the fathers had or more eight years of education, and nearly 50% of the mothers were employed. Only 112 children (23.68%) had visited the dentist; of those, 67.77% went for preventive reasons. Most (63.11%) used public services. The prevalence of dental caries was 33.7% (dmft ≥ 1), and only 29 filled surfaces were observed in 11 teeth. No missing teeth were observed (Table 1).

**Table 1:** Sociodemographic and clinical characteristics of the sample

**Variable**	**N**	**%**
***Child’s gender***		
Male	232	48.54
Female	246	52.46
***Child’s age (months)***		
12–35	186	38.91
36–59	292	61.09
***Child’s skin color***		
White	379	79.29
Non-White	99	20.71
***Household income***		
>1 BMW*	338	74.78
≤1 BMW*	114	25.22
***Mother’s level of education***		
≥8 years	268	56.78
<8 years	204	43.22
***Father’s level of education***		
≥8 years	247	55.38
<8 years	199	44.62
***Does the child brush his/her teeth?***		
Yes	434	90.99
No	43	9.01
***Children’s dental caries***		
dmft = 0	317	66.32
dmft ≥ 1	161	33.68
***Children’s previous visit to the dentist***		
Yes	112	23.68
No	361	76.32
***Reason for dental visit***		
Preventive	82	67.77
Others than preventive	39	32.23
***Type of healthcare system***		
Private	45	36.89
Public	77	63.11

*BMW* Brazilian minimum wage; values lower than 478 due to missing data.

Table 2 shows that the prevalence of children who had never visited a dentist was associated with children’s age, maternal education, and frequency of tooth brushing. These associations remained significant in the multiple regression analysis. Older children were more likely than younger children to use dental services. In addition, children whose mothers had less than eight years of education were 13% more likely (PR: 1.13, 95% CI: 1.02-1.24) to have never visited a dentist, in comparison to children whose mothers had eight or more years of education. Further, children who did not brush their teeth regularly were less likely to have visited the dentist than children who did (PR: 1.16, 95% CI: 1.05-1.27).

**Table 2:** Child’s dental visit and associated factors (prevalence ratio: 95% CI)

**Variables**	**Have never gone to the dentist**	
	**PR (95% CI)**	**PR**_**adj. **_**(95% CI)**
***Child’s gender***	*p* = 0.17	**
Male	1	
Female	1.07 (0.97–1.17)	
***Child’s age (months)***	*p* < 0.001	*p <* 0.001
12–35	1	1
36–59	0.84 (0.76–0.92)	0.84 (0.76–0.93)
***Child’s skin color***	*p* = 0.56	**
White	1	
Non-White	0.96 (0.84–1.10)	
***Household income***	*p* = 0.14	**
>1 BMW*	1	
≤1 BMW*	1.08 (0.97–1.21)	
***Mother’s level of education***	*p* = 0.02	*p =* 0.02
≥8 years	1	1
<8 years	1.09 (1.02–1.25)	1.13 (1.02–1.24)
***Father’s level of education***	*p* = 0.10	**
≥8 years	1	
<8 years	1.09 (0.98–1.21)	
***Does child brush teeth?***	*p* = 0.00	*p =* 0.00
Yes	1	1
No	1.28 (1.17–1.40)	1.16 (1.05–1.27)
***Children’s dental caries***	*p* = 0.15	**
dmft = 0	1	
dmft ≥ 1	0.92 (0.82–1.03)	
***Parents’ perception of child’s oral health***	*p* = 0.38	**
Good/excellent	1	
Fair/poor	0.91 (0.74–1.12)	

*p* Wald statistics, *BMW* Brazilian minimum wage, *PR* prevalence ratio, *PR*_*adj*_ adjusted prevalence ratio; *95% CI* 95% confidence interval. **Variables not fitted in the final multiple model after the adjustment.

The association between the use of dental care services for non-preventive reasons and predictor variables is shown in Table 3. Low income, the presence of caries, and poor parent-perceived child oral health were associated with the prevalence of dental care use for treatment reasons even after adjustment for other covariates. Children from low-income families were more likely to have visited the dentist for treatment rather than preventive reasons (PR: 1.67, 95% CI: 1.05–2.66). Children with dental caries used dental care services for non-preventive reasons 2.37 times more often than their counterparts without caries. Moreover, the probability of having visited the dentist for non-preventive reasons was 1.70 times higher for children with “poor” parent-perceived oral health compared to those with “good” parent-perceived oral health.

**Table 3:** Reason for the dental visit and associated factors (prevalence ratio: 95% CI)

**Variables**	**Reason for the visit (other than preventive)**	
	**PR (95% CI)**	**PR**_**adj. **_**(95% CI)**
***Child’s gender***	*p* = 0.67	**
Male	1	
Female	0.89 (0.53–1.52)	
***Child’s age (months)***	*p* = 0.16	**
12–35	1	
36–59	1.73 (0.81–3.73)	
***Child’s skin color***	*p* = 0.21	**
White	1	
Non-White	1.40 (0.8–2.41)	
***Household income***	*p* = 0.00	*p* = 0.03
>1 BMW*	1	1
≤1 BMW*	0.71 (0.57–0.89)	1.67 (1.05–2.66)
***Mother’s level of education***	*p* = 0.33	**
≥8 years	1	
<8 years	1.29 (0.77–2.17)	
***Father’s level of education***	*p* = 0.13	**
≥8 years	1	
<8 years	0.62 (0.34–1.15)	
***Does child brush teeth?***	*p =* 0.08	**
Yes	1	
No	2.13 (0.91 – 4.96)	
***Children’s dental caries***	*p* = 0.00	*p* = 0.01
dmft = 0	1	1
dmft ≥ 1	2.98 (1.67–5.33)	2.37 (1.31–4.30)
***Parents’ perception of child’s oral health***	*p* = 0.00	*p* = 0.02
Good/excellent	1	1
Fair/poor	2.88 (1.79–4.63)	1.70 (1.07–2.70)

*p* Wald statistics, *BMW* Brazilian minimum wage, *PR* prevalence ratio, *PR*_*adj*_ adjusted prevalence ratio; *95% CI* 95% confidence interval. **Variables not fitted in the final multiple model after the adjustment.

## Discussion

We assessed the association between the use of dental care services and various psychosocial and socioeconomic variables. Overall, our results demonstrated that a high proportion of preschool children in Brazil had never visited a dentist, and that psychosocial and socioeconomic variables were significant predictors of dental service utilization.

The low use of oral healthcare services in our study (23.68%) was similar to that found by Ardenghi [2] in the same population in 2010, but higher than that found by Kramer et al. [9], who reported that only 13.3% of their sample of children had already consulted a dentist. Our results showed that a larger proportion of older than younger children used dental services. These findings support those of previous studies, and can be attributed to the cumulative effect of oral problems as children grow. Another explanation for this phenomenon is insufficient knowledge about the importance of early preventive dental care [2,15,16]. It is important to investigate whether this is associated with parents’ perception of the need for a preventive appointment, or whether parents only bring their children to the dentist following the emergence of symptoms or presence of oral health problems [9,17].

Socioeconomic status plays an important role in the utilization of health services [4,9,17,18]. Maternal education was associated with the use of dental services, indicating that lower knowledge of oral health leads to unhealthy behaviors and less interest in preventive treatment [2,4]. Education can lead people to be more health-conscious, and helps them make better and healthier lifestyle choices [19].

This study showed that children who did not brush their teeth were less likely to regularly visit the dentist than those who did; this can be explained by the absence of a preventive dental healthcare policy [9]. However, one may argue that the relationship could be considered in the inverse direction. In fact, children who did not visit the dentist were found to have unhealthy behaviors regarding tooth brushing.

Mothers’ perception of their child’s OHRQoL was associated with the utilization of dental services for treatment, confirming the notion that greater oral health need (perceived or normative) is an important predictor of the use of dental health services in preschool children [17,20]. This is in agreement with observations by Sohn [21]. Caregivers’ unfavorable perception of their children’s oral health motivates them to seek dental care for them [17]. The presence of untreated dental caries in children is associated with parents’ perception that their children’s oral health is poor, irrespective of their socioeconomic status [10,22,23]. Thus, a poor parental perception of children’s health can be used as a measure of dental care need.

Dental visits for non-preventive reasons are directly related to the presence of dental caries. The utilization of dental services by children and adolescents is often driven by the presence of pain [10,24], which is a consequence of untreated dental caries.

Data from this study must be assessed with caution. Our study employed a cross-sectional design, which pre-empts inferences regarding causality and temporal relationships between variables; thus, longitudinal studies should be conducted to investigate this issue. The possibility of recall bias is also a concern when working with questionnaires; however, the effect of this bias is not expected to be significant since self-reported dental care has been found to be a valid measure of dental care use across different socioeconomic strata [25]. In addition, one could argue that we did not use a validated questionnaire to measure the children’s oral health-related quality of life. However, studies have shown that the single-item perceived oral health rating is related to other self-reported measures of oral health, such as multi-item indicators [26]. Moreover, this methodology was used in a previous study and is considered valid [27]. Thus, a single-item rating of perceived oral health is particularly appropriate for obtaining information from children’s parents. All respondents to our questionnaire were parents, but we have no exact data on the relative proportion of mothers and fathers. However, as more than 90% of respondents were mothers, we believed that this issue did not influence our results.

## Conclusions

In conclusion, this study demonstrated that psychosocial and socioeconomic factors are important predictors of the use of dental care services. Public health policy-makers should assess these variables and devote resources to eliminate the sources of this inequity in the use of dental services, thereby improving population health.

## Abbreviations

OHRQoL: Oral health-related quality of life; WHO: World Health Organization; ECOHIS: Early childhood impact scale; PR: Prevalence ratio; CI: Confidence interval.

## Competing interests

The authors declare that they have no competing interests.

## Authors’ contributions

RMV, BAA, CRST, ST and CP assisted in data collection and writing the manuscript. TMA and FMM performed all statistical analyses, revised the manuscript, and supervised the study. All authors have read and approved the final manuscript.

## Pre-publication history

The pre-publication history for this paper can be accessed here:

http://www.biomedcentral.com/1472-6831/13/60/prepub
